# Brain-Computer Interfaces in Rehabilitation: Implementation Models and Future Perspectives

**DOI:** 10.7759/cureus.88873

**Published:** 2025-07-28

**Authors:** Raktim Swarnakar

**Affiliations:** 1 Physical Medicine and Rehabilitation, All India Institute of Medical Sciences, New Delhi, New Delhi, IND

**Keywords:** artificial intelligence and education, brain computer interface, neurorehab, physical medicine and rehabilitation, rehab

## Abstract

Brain-computer interfaces (BCIs) represent an emerging advancement in rehabilitation, enabling direct communication between the brain and external devices to aid recovery in individuals with neurological impairments. BCIs can be classified into invasive, semi-invasive, non-invasive, or hybrid types. By interpreting neural signals and converting them into control commands, BCIs can bypass damaged pathways, offering therapeutic potential for conditions such as stroke, spinal cord injury, traumatic brain injury, and neurodegenerative diseases such as amyotrophic lateral sclerosis. BCIs' current applications, such as motor restoration via robotic exoskeletons and functional electrical stimulation, cognitive enhancement through neurofeedback and attention training, and communication tools for individuals with severe physical limitations, are largely being explored within research settings and are not yet part of routine clinical practice. Advances in EEG signal acquisition, machine learning, wearable and wireless systems, and integration with virtual reality are enhancing the clinical utility of BCIs by improving accuracy, adaptability, and usability. However, widespread clinical adoption faces challenges, including signal variability, training complexity, data privacy, and ethical and regulatory issues. Ethical challenges in BCI include issues related to the ownership and misuse of brain data, risks of neural interference, threats to autonomy and personal identity, as well as concerns around data privacy, user consent, emotional manipulation, and accountability in neural interventions. In this context, this editorial has also proposed one model (NEURO model checklist) for BCI implementation in rehabilitation. The future of BCIs in rehabilitation lies in developing personalized, closed-loop, and home-based systems, enabled by interdisciplinary collaboration among clinicians, engineers, neuroscientists, and policymakers. With continued research and ethical implementation, BCIs have the potential to transform neurorehabilitation and greatly enhance patient outcomes and quality of life.

## Editorial

In recent years, brain-computer interfaces (BCIs) have emerged as a promising tool in rehabilitation [1]. BCIs are systems that enable direct communication between the brain and external devices, bypassing damaged neural pathways [2]. By decoding neural signals and translating them into actionable commands, BCIs can facilitate motor control, communication, and cognitive engagement, making them especially valuable in neurorehabilitation [3,4]. The integration of BCIs into rehabilitation programs marks a shift toward more personalized, interactive, and neuroplasticity-driven therapies, offering a transformative future for patients and clinicians alike.

BCIs have evolved significantly since their inception in early neuroscience experiments of the 1960s; however, despite technological advances, their clinical applications remain limited and are still largely in the experimental stage [2]. Initial breakthroughs demonstrated that brain signals could control external devices, leading to the development of both invasive and non-invasive systems [2]. Over the decades, improvements in signal acquisition, processing algorithms, and device integration have made BCIs more practical and relevant in real-world rehabilitation settings [2,3].

BCI enables direct communication between the brain and external devices, translating brain signals into control commands [2]. The key components include signal acquisition (e.g., electroencephalogram (EEG), electrocorticography (ECoG)), signal processing, translation algorithms, output devices, and feedback systems [2]. BCIs are classified as invasive, non-invasive, or hybrid. Invasive BCIs offer high resolution but involve surgical risks [2]. BCIs function through signal generation, detection, processing, translation, and feedback, forming a closed-loop system ideal for promoting neuroplasticity and functional recovery [1,4].

BCIs have evolved into clinical tools for neurorehabilitation [1]. Studies showed that BCI-assisted therapy enhances motor recovery when combined with conventional therapy [3-5]. Cognitive rehabilitation is another area of BCI application [2]. These systems monitor attention, memory, and executive function, using EEG signals to adapt cognitive tasks [2]. For patients with severe disabilities, such as amyotrophic lateral sclerosis (ALS) or locked-in syndrome, BCIs serve as communication aids using P300 spellers (BCI application designed to communicate language by detecting event related potentials in a person's EEG signal), steady-state visually evoked potential (SSVEP) systems (BCI that uses the brain's response to visual stimuli flickering at specific frequencies to enable communication and control), or motor imagery to select commands [2]. Despite these advancements, several challenges continue to hinder the widespread clinical adoption of BCIs. Current challenges in BCI implementation include poor signal quality due to physiological and environmental noise, limited spatial resolution of non-invasive hardware, user discomfort during prolonged use, the requirement for subject-specific training, high costs, and significant ethical concerns related to data privacy and neural modulation [2].

In a recent systematic review on visual evoked potential-based BCIs for motor rehabilitation applications, only 7.77% of the studies were focused on motor rehabilitation, and just four of these conducted tests involving patients. Of the 34 included studies, 26.47% used P300 signals, while 55.8% used SSVEP signals [5]. Another recent systematic review focusing on BCI systems for upper and lower limb rehabilitation identified 11 studies targeting upper limbs, six targeting lower limbs, and one addressing both. It was also noted that six studies used combined visual and auditory feedback, while four incorporated fully immersive virtual reality (VR) environments. These studies reported benefits such as improved training outcomes, cost-effectiveness, and enhanced user motivation, and highlighted the need for continued development of user-accessible interfaces with integrated feedback mechanisms [3].

Technological advances have driven BCI development, with EEG remaining the most common non-invasive technique - now enhanced by high-density arrays, dry electrodes, and artifact-reduction algorithms [2]. Future BCI applications focus on personalized therapy, robotic exoskeleton integration, artificial intelligence, and immersive VR environments [3,5]. Personalized BCIs adapt to individual brain patterns and therapy goals [2]. Robotic systems provide motor assistance and feedback, while VR/augmented reality (VR/AR) enhances task realism [3,5].

Ethical and practical issues must be addressed. Brain data is profoundly sensitive, calling for more than just privacy safeguards and informed consent. As neurotechnologies advance, critical ethical issues emerge, such as who owns the data generated from one’s brain, and how it might be accessed or misused. The possibility of unauthorized interference with neural systems ("brain hacking") introduces new security concerns. Moreover, technologies that influence behavior or perception may challenge an individual’s ability to make autonomous decisions. At a deeper level, these interventions can blur the lines of personal identity, raising complex questions about agency and selfhood in the age of BCIs. Regulatory challenges may persist due to limited long-term data and evolving technologies. For BCIs to transition from experimental tools to mainstream clinical technologies, clear regulatory approval pathways and well-designed, large-scale clinical trials are essential. These trials must go beyond demonstrating technical feasibility to rigorously evaluate long-term safety, reliability, and real-world effectiveness. Safety concerns include risks of surgical implantation (where applicable), device malfunction, unintended neural effects, and psychological consequences such as dependency or altered cognition. Transparent standards for testing, reporting, and monitoring are critical to building trust among clinicians, patients, and the public and to ensuring that innovation does not outpace ethical and medical safeguards. Non-invasive signals are prone to noise; improving sensors and algorithms is vital [2]. Non-invasive neural signals, such as those captured via EEG, are highly susceptible to various sources of noise, including muscle activity (electromyography, or EMG), eye movements (electrooculography, or EOG), and environmental electrical interference. Enhancing signal quality requires both hardware improvements, such as better shielding and dry electrode design, and algorithmic advances, such as artifact rejection, adaptive filtering, and machine learning-based signal decoding techniques that can distinguish true neural patterns from noise. Clinician training and cross-disciplinary coordination are key to effective BCI deployment [5].

**Figure 1:**
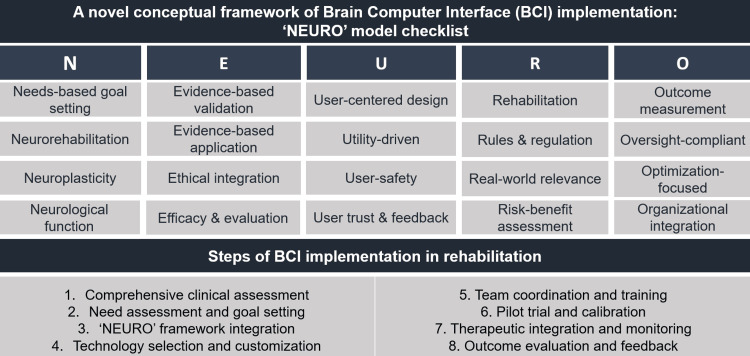
A novel conceptual framework of brain computer interface (BCI) implementation ("NEURO" model) in rehabilitation. Credit: The author

This editorial also hereby proposes the "NEURO" framework as a structured approach to guide the clinical translation of BCIs into neurorehabilitation (Figure 1).

It emphasizes the integration of technological innovation with patient-centered care, ethical responsibility, and regulatory compliance to optimize therapeutic outcomes. By focusing on clinical needs, robust scientific evidence, user-centered design, regulatory and ethical alignment, and neuroplasticity-driven goals, the "NEURO" framework may serve as a practical and forward-looking guide for the effective implementation of BCIs in routine rehabilitation settings.

In conclusion, while still largely in the experimental stage, BCIs hold the potential to become a transformative approach in rehabilitation, offering a promising bridge between medicine and engineering to restore function and independence. Despite existing challenges, continued innovation, rigorous validation, and ethically guided deployment have the potential to transform BCIs into powerful tools that make neurorehabilitation more personalized, accessible, and effective for diverse patient populations.
